# Genetic Variation of Drought Tolerance in *Pinus pinaster* at Three Hierarchical Levels: A Comparison of Induced Osmotic Stress and Field Testing

**DOI:** 10.1371/journal.pone.0079094

**Published:** 2013-11-01

**Authors:** Maria João Gaspar, Tania Velasco, Isabel Feito, Ricardo Alía, Juan Majada

**Affiliations:** 1 Departamento de Genética e Biotecnologia, Universidade de Trás os Montes e Alto Douro, Vila Real, Portugal; 2 Centro de Estudos Florestais, Instituto Superior de Agronomia, ULisboa, Tapada da Ajuda, Lisboa, Portugal; 3 Sección Forestal, SERIDA, Finca Experimental La Mata, Principado de Asturias, Spain; 4 Department of Forest Ecology and Genetics, INIA, Forest Research Centre, Madrid, Spain; 5 Sustainable Forest Management Research Institute, University of Valladolid-INIA, Palencia, Spain; 6 Sección Forestal, CETEMAS, Finca Experimental La Mata, Principado de Asturias, Spain; Universidade Federal do Rio de Janeiro, Brazil

## Abstract

Understanding the survival capacity of forest trees to periods of severe water stress could improve knowledge of the adaptive potential of different species under future climatic scenarios. In long lived organisms, like forest trees, the combination of induced osmotic stress treatments and field testing can elucidate the role of drought tolerance during the early stages of establishment, the most critical in the life of the species. We performed a Polyethylene glycol-osmotic induced stress experiment and evaluated two common garden experiments (xeric and mesic sites) to test for survival and growth of a wide range clonal collection of Maritime pine. This study demonstrates the importance of additive *vs* non additive effects for drought tolerance traits in *Pinus pinaster*, and shows differences in parameters determining the adaptive trajectories of populations and family and clones within populations. The results show that osmotic adjustment plays an important role in population variation, while biomass allocation and hydric content greatly influence survival at population level. Survival in the induced osmotic stress experiment presented significant correlations with survival in the xeric site, and height growth at the mesic site, at population level, indicating constraints of adaptation for those traits, while at the within population level no significant correlation existed. These results demonstrate that population differentiation and within population genetic variation for drought tolerance follow different patterns.

## Introduction

Under the on-going climate change scenario, longer, more frequent and more intense drought periods are expected in south-western Europe [1], and water stress will therefore be a leading constrain on plant survival and productivity [2]. Forest tree populations are thus facing new selection pressures and might be unable to track their bioclimatic envelope [3] over the time scale at which these changes are occurring. There is a strong debate about the potential to adapt to these new environmental conditions, which involves phenotypic plasticity at the individual level, and either genetic adaptation or migration at the population level [4]. But in order to evaluate this possibility, it is necessary to characterize the adaptive genetic variation within forest tree species [5] at different hierarchical levels, particularly at early ages. The first years are the most critical phase for the establishment of forest species since seedlings are extremely susceptibility to resource limitations and it is at this stage that most mortality occurs, playing an important role in natural regeneration [6]. The importance of genetic adaptation in forest trees is stressed by the fact that genetic differentiation between populations and clinal variation along environmental gradients are very common in forest tree species (90% and 78% respectively) and, thus, responding to climate change will likely require that the quantitative traits of populations again match their environments [7]. 

Nonetheless, we have to consider that drought tolerance in *Pinus* species involves various processes and mechanisms. The adjustment of pine's hydraulic system to local climatic conditions occurs primarily through modifications of shoot radial growth and direct stomatal control [8]. In this sense, species adapted to xeric conditions, but with higher growth rates are vulnerable to drought-induced decline, and after consecutive severe droughts a reduction in basal area increment can be observed [9]. Moreover, another strategy, osmotic adjustment, has been cited as a mechanisms underlying adaptation to drought [10], with higher solute accumulation occurring in the provenances from drier sites. Additionally, different allocation of resources among shoots and roots have been invoked as drought tolerance mechanisms in pines [11–13]. Drought stress episodes can be simulated in *Pinus* with methods based on the use of an osmoticum, such as polymers with high molecular weight (i.e. Polyethylene glycol, PEG) [14–16]. The use of PEG allows the analysis of short and long term responses, ensuring the standardization of the level of water stress perceived by the plant [14], and assures a more homogeneous water stress than by controlling the water supply through different watering regimes. This osmotic is known to have low toxicity due to its inability to penetrate pores in the cell wall and therefore inside tissues [17,18]. Also, by imposing water stress on plants in hydroponic culture, we are able to observe the effects of water stress on plants in the absence of the physical constrains that occur in drying soils [14]. This methodology has already been studied in relation to early selection [19], but it is necessary to asses if the results of PEG-induced osmotic stress tests are indeed associated with fitness related traits measured in the field. 

Maritime pine (*Pinus pinaster* Ait.) is a highly valuable Mediterranean coniferous species that grows under contrasting water availabilities (high in the Atlantic coasts of Portugal, Spain and France and much lower in continental Spain and the Mediterranean coasts of Southern Europe and North of Africa) [20]. Throughout its natural geographic distribution, this species exhibits a high level of population differentiation for survival [21], growth [22–26] phenology, and drought tolerance [14,27–29]. It is considered highly plastic, whereby some provenance or genotypes have a superior performance over a wide range of conditions, and it has been demonstrated that sensitivity to drought varies between provenances [27,28,30,31]. *P. pinaster* populations from mesic origins show higher phenotypic plasticity than the xeric ones for drought related traits. They adapt to the xeric conditions and show similar or even better performance than the xeric populations for drought related traits [12,32]. The pattern of variation differs between drought related traits (e.g. water use and hydraulic efficiency and biomass allocation), presenting either uniform or divergent selection [20,33]. 

Studies that consider different hierarchical levels of variation such as population, family and genotypes within families, are essential to analyze tradeoffs among traits that can determine the evolutionary trajectories in populations [34]. Genetic correlations, genetic variance, and cross-environmental genetic correlations would determine the expected response to selection on trees or families within the populations [35]. Such an approach would, though, allow the exploration of a multi-dimensional space of traits (e.g. WUE, osmotic potential, root/shoot allocation, growth phenology) under severe drought stress. Also, it would allow the estimation of the magnitude of the difference between additive and non-additive effects in drought tolerance traits. Until now, the main constraint to achieving this goal has been the lack of precise and standardized high-throughput phenotyping technologies, something that would require close collaboration between geneticists, breeders, physiologists and ecologists [36], as well as a suitable method to efficiently produce the clonal material to be used in testing [37].

The main objectives of this work are, firstly, to assess genetic variability in a PEG-induced osmotic stress experiment of Maritime pine at three hierarchical levels: population, family and genotype and, secondly, to check the correlations of these results with fitness proxies in contrasting field conditions.

The obtained results allows determining the efficiency of PEG as an early evaluation test for drought tolerance in Maritime pine, also this study allows the exploration of evolutionary trajectories of traits when submitted to severe drought stress. 

## Results

### Population variation

**Figure 1:**
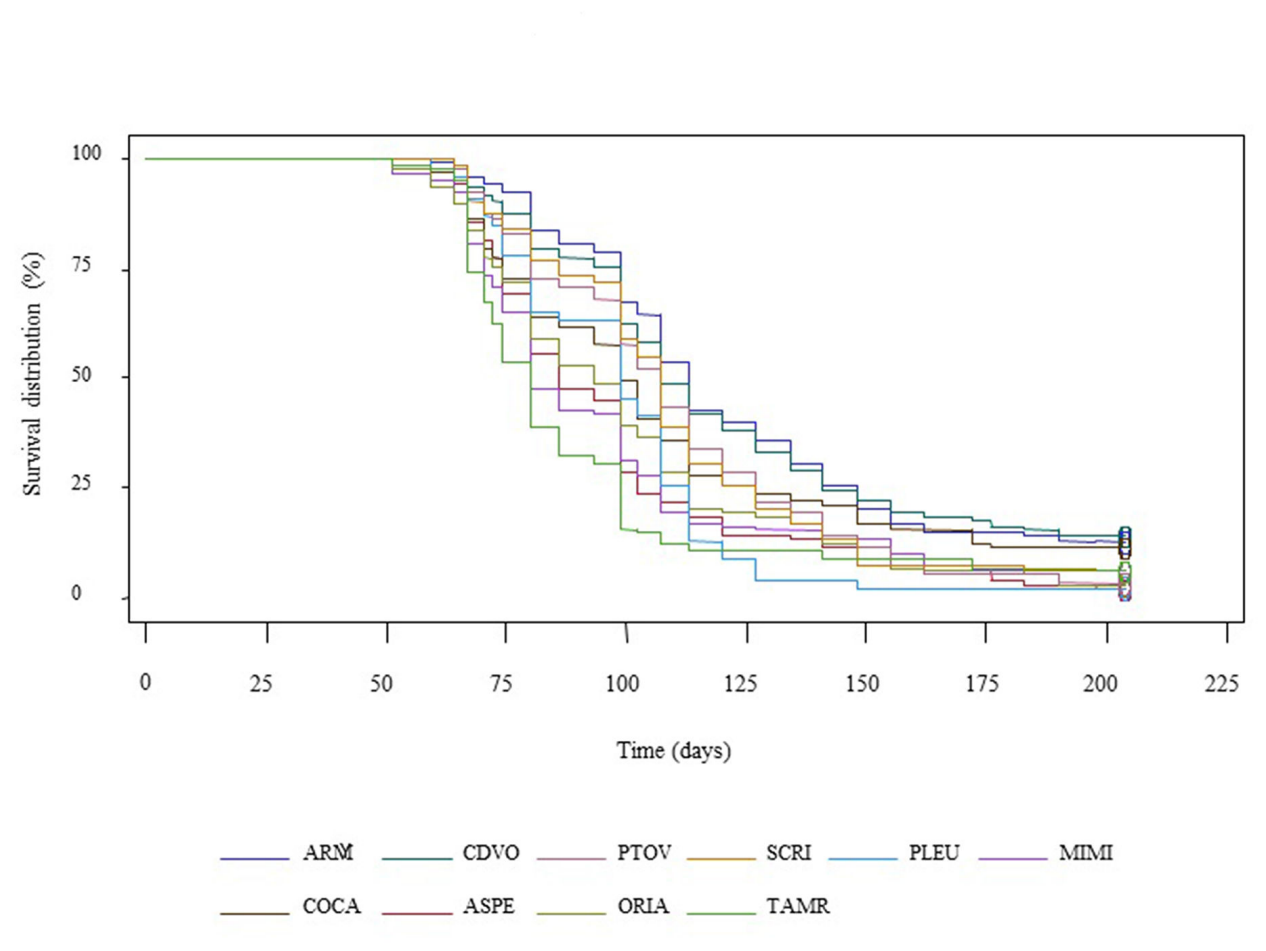
Distribution function of survival by provenance during the PEG-induced osmotic stress experiment, estimated by Kaplan-Meier method.

The results of survival rates of provenances in the controlled condition experiment, based on Kaplan-Meier estimations, are presented in Figure 1. Applying both log-rank test, and the Wilcoxon test it was possible to establish the ranking of survival between populations, and significant differences (α<0.0001) were observed between the survival rates of the different provenances in the PEG-induced osmotic stress experiment (Table S1). 

The most discriminant time lapse was between 90 and 110 days, and based on this result we calculated the survival not at the end of the experiment but at 100th day (S100). 

Among all provenances TAMR was the first to reach T50, after 60.7 days of trial, followed by MIMI, COCA, ASPE and ORIA (Table 1). On the other hand, CDVO, ARMY, PTOV and SCRI - all provenances from Atlantic Spain - presented values of T50 over 100 days. A similar pattern can be observed for survival at 100th day, where the provenances from Atlantic Spain (ARMY, CDVO; PTOV, SCRI) presented the highest values, followed by Central and Southern Spanish populations (ASPE, COCA, ORIA). The provenance with the lowest survival rate was the Moroccan population (TAMR). 

**Table 1:** Descriptive statistics at the population level for each of the measured traits (Standard errors in brackets. Codes of populations in Table 4. Means with the same letter do not differ significantly, α<0.05).

**Trait**	**ARMY**	**ASPE**	**CDVO**	**COCA**	**MIMI**	**ORIA**	**PLEU**	**PTOV**	**SCRI**	**TAMR**
**T50**	100.5 (4.40)	85.95 (4.44)	108.86 (4.47)	84.59 (4.79)	82.63 (4.16)	86.68 (4.44)	95.67 (7.90)	104.18 (4.44)	97.98 (4.60)	60.72 (4.10)
	a b	b c	a	c	c	b c	a b c	a b	a b c	d
**S100**	59.60 (3.62)	39.68 (3.70)	67.93 (3.62)	49.44 (3.79)	38.89 (3.46)	43.65 (3.70)	46.83 (6.41)	62.80 (3.54)	57.32 (3.62)	15.97 (3.46)
	a b	d	a	c d	d	d	c d	a b	b c	e
**M_HT**	17.82 (0.45)	19.24 (0.48)	19.92 (0.49)	18.47 (0.49)	17.51 (0.53)	15.67 (0.43)	19.49 (0.77)	18.99 (0.51)	20.49 (0.60)	14.84 (0.48)
	b c d	a b c	a b	a b c	c d	d e	a b c	a b c	a	e
**X_HT**	14.75 (0.67)	16.75 (0.78)	16.94 (0.74)	15.71 (0.74)	16.23 (0.74)	13.75 (0.72)	17.89 (0.80)	17.33 (0.57)	19.34 (0.70)	15.08 (0.71)
	b c	b	a b	b c	b c	c	a b	a b	a	b c
**M_SV**	84.52	82.51	80.43	86.36	79.17	81.88	87.5	86.36	89.13	72.22
	b	b	a b c	b	a b c	b	b	b	a b c	a
**X_SV**	66.86	72.82	61.41	63.64	60.82	61.49	68.25	59.77	68.48	60.41
	a c	a	a b	c a	a b	a b	c	a b	c	a b
**LWC**	60.90 (1.19)	60.66 (1.35)	59.22 (1.09)	61.05 (1.20)	56.01 (3.07)	63.14 (1.36)	54.35 (5.53)	61.03 (1.92)	62.69 (1.20)	72.10 (6.38)
	b c	b c	b c	b c	b c	b	c	b c	a b c	a
**RWC**	89.74 (0.014)	91.39 (0.016)	86.65 (0.013)	86.96 (0.014)	84.35 (0.036)	88.89 (0.016)	66.99 (0.066)	86.94 (0.023)	93.28 (0.014)	96.16 (0.076)
	a b	a b	b	a b	b	a b	c	a b	a b	a
**SBR**	0.59 (0.014)	0.64 (0.023)	0.62 (0.012)	0.58 (0.014)	0.63 (0.022)	0.54 (0.023)	0.66 (0.041)	0.58 (0.014)	0.59 (0.015)	0.53 (0.047)
	a	a	a	a b	a b	a b	a b	a b	a	c
**ROB**	0.460 (0.034)	0.26 (0.055)	0.41 (0.028)	0.42 (0.035)	0.30 (0.055)	0.49 (0.057)	0.27 (0.099)	0.40 (0.034)	0.38 (0.036)	0.22 (0.114)
	a b	b	a b	a b	a b	a	a b	a b	b	b
**TB**	1.09 (0.076)	0.74 (b.119)	1.09 (0.061)	0.98 (0.076)	0.79 (0.119)	1.11 (0.124)	0.81 (0.214)	0.98 (0.075)	0.92 (0.077)	0.49 (0.248)
	a b	b	a b	a b	a b	a	a b	a b	a b	c

**T50**: days to reach 50% mortality, PEG-induced osmotic stress experiment, **S100**: Survival at 100th day, PEG-induced osmotic stress experiment, **M_HT**: Total Height (mm) at the Mesic site, at age 1 after planting, **X_HT**: Total Height (mm) at the Xeric site, age 1 after planting, **M_SV**: Survival (%) at Mesic site, age 1 after planting, **X_SV**: Survival (%) at Xeric site, age 1 after planting, **LWC**: Leaf Water Content (%), PEG-induced osmotic stress experiment, **RWC**: Relative Water Content, PEG-induced osmotic stress experiment, **SBR**: Shoot biomass ratio (g), PEG-induced osmotic stress experiment, **ROB**: Root Biomass (g), PEG-induced osmotic stress experiment, **TB**: Total Biomass (g), PEG-induced osmotic stress experiment.

In the Mesic site the provenances with greater height were SCRI, CDVO, and PLEU (20.5, 19.9 and 19.5 cm respectively), while in the Xeric site, ORIA had the lowest value (13.7 cm). SCRI, with a height of 19.3 cm, was the provenance that displayed the higher value also in the xeric trial. Some of the Spanish and French Atlantic populations presented a high survival in the Mesic (SCRI, PLEU) and Xeric trials (ASPE, SCRI and PLEU), while TAMR presented the lowest survival in the two field trials. 

**Figure 2:**
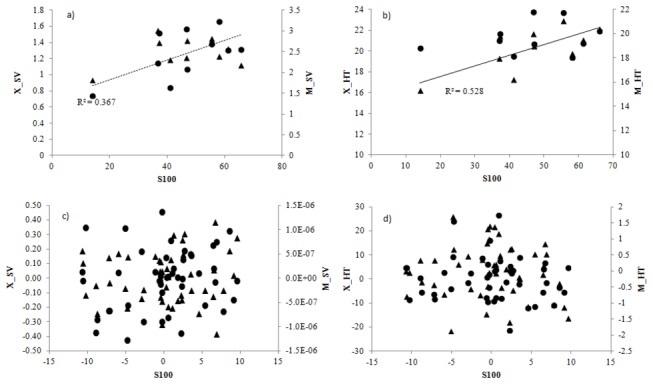
Regressions between provenance BLUE’s (a, b) and family BLUP’S (c, d) of S100 with Height (b, d) and Survival (a, c) evaluated in the two field experiments (circle: Xeric Conditions, triangle: Mesic conditions).

Significant correlations were found between some traits (Table 2). T50 and S100 were highly correlated (0.98±0.02, α<0.001) and both T50 and S100 were significantly correlated with M_HT (α<0.001) and X_SV (α<0.05) (Figure 2). Moreover, there was a high degree of correlation between height measured in the two sites (0.73±0.154; α<0.001) which indicates a consistence in the behaviour of populations in distinct environments.

**Table 2:** Correlation coefficient between Provenances BLUE’s values in lower triangle and Genetic correlations in upper triangle (Additive upper value, Genetic lower value in bold).

	**T50**	**S100**	**M_HT**	**X_HT**	**M_SV**	**X_SV**
**T50**	0.314±0.155	0.83±0.04***	-0.21±0.14ns	-0.14±0.14ns	-	0.17±0.14 ns
	0.398 ±0.148	0.79±0.02***	-0.13±0.07*	-0.08±0.07ns	0.05±0.07ns	0.16±0.06*
**S100**	0.98±0.02***	0.356±0.158	-0.09±0.14ns	-0.10±0.14 ns	-	-0.10±0.14 ns
		0.479±0.149	-0.08±0.07ns	-0.05±0.07ns	0.07±0.07ns	0.12±0.07ns
**M_HT**	0.75±0.14***	0.73±0.16***	0.232±0.113	0.53±0.10 ***	-	0.03±0.14 ns
			0.366±0.105	0.17±0.06*	0.14±0.07*	0.03±0.07ns
**X_HT**	0.31±0.30 ns	0.22±0.31ns	0.73±0.15***	0.261±0.124	-	0.20±0.14ns
				0.376±0.115	0.06±0.07ns	0.08±0.07ns
**M_SV**	0.23±0.316 ns	0.26±0.31 ns	0.49±0.253 ns	0.24±0.31 ns	-	0.22±0.14ns
					0.025a	0.12±0.07ns
**X_SV**	0.70±0.171*	0.61±0.21ns	0.75±0.14***	0.43±0.27 ns	0.33±0.30 ns	0.293a
						0.406a

Heritability in diagonal (narrow sense upper value, broad sense lower value), for fitness related traits in the PEG-induced osmotic stress experiment and the two field experiments. Standard errors of estimations are included.

Abbreviations as in Table 1. ^a^ heritabilities adjusted to the liability scale. Statistical significance: * α < 0.05, ** α < 0.01; *** α < 0.001; ns: non significant.

In general, correlation coefficients between traits evaluated in the PEG-induced osmotic stress experiment were stronger at population level (Table 3). T50 and S100 showed a positive correlation with total biomass (α<0.05). However, only S100 presented a positive correlation with ROB (α<0.05), while T50 was negatively correlated with LWC (α<0.05).

**Table 3:** Correlation coefficients between traits in the PEG-induced osmotic stress experiment.

	**Population**			**Additive**		**Genetic**	
	**T50**	**S100**	**T50**		**S100**	**T50**	**S100**
**LWC**	- 0.67±0.18 *	-0.60±0.21	0.07±0.14 ns		-0.15±0.14 ns	0.04±0.10 ns	0.08±0.10 ns
**RWC**	-0.46±0.26 ns	-0.35±0.29 ns	0.38±0.12 **		0.33±0.13 *	0.15±0.10 *	0.08±0.10 ns
**SBR**	0.45±0.27 ns	0.30±0.30 ns	0.19±0.14 ns		-0.03±0.14 ns	0.05±0.13 ns	-0.02±0.13 ns
**ROB**	0.55±0.23 ns	0.66±0.19 *	0.07±0.14 ns		0.18±0.14 ns	0.09±0.13 ns	0.14±0.13 *
**TB**	0.82±0.11 **	0.85±0.09 **	0.05±0.14 ns		0.09±0.1 ns4	0.07±0.13 ns	0.10±0.13 ns

Standard errors of estimations are included.

Abbreviations as in Table 1. Statistical significance: * α < 0.05, ** α < 0.01; *** α < 0.001; ns: non significant.

### Additive and total within-population genetic variation

The various fitness related traits showed significant levels of genetic variation within populations (Table S2), with significant heritabilities (narrow and broad sense), except for M_SV where no significant differences (p<0.05) were observed between families (Table 2). 

Comparing narrow sense and broad sense heritabilities for all traits, non-additive effects (although statistically significant, Table S2) were of scant importance for the measured traits related to drought tolerance in *P pinaster*. S100 and T50 were the traits with the highest heritabilities (h^2^=0.36±0.158; H^2^=0.48±0.149 and h^2^=0.31±0.155; H^2^=0.40±0.148 respectively). Survival in the Mesic site displayed the lowest heritability values (H^2^=0.03), while in the Xeric site presented higher heritabilities (h^2^=0.29 and H^2^=0.41).

The genetic architecture of the traits (indicated by the structure of genetic correlations), differed to that observed between populations. Highly significant positive additive and total genetic correlations were observed between T50 and S100 (0.83± 0.04 and 0.79± 0.02 respectively; α<0.001) (Table 2), but only T50 presented a significant genetic correlation with M_HT and X_SV (α<0.05). Significant additive and genetic correlations were detected for Height between the two field sites (0.53±0.10, α<0.001 and 0.17±0.06, α<0.01, respectively).

It is interesting to notice that the additive and total genetic correlations between T50/S100 and hydric and biomass traits measured in the PEG-induced osmotic stress experiment do not follow the same pattern as the correlations at the population level (Table 3). Positive additive correlations were detected between RWC and T50 and S100 (α<0.05), while the remaining additive correlations were not significant. Regarding total genetic correlations those between T50 and RWC, and S100 and ROB, were the only two significant (α<0.05). 

## Discussion

To date, some studies have been performed in young plants of *P. pinaster* which aim to study survival in conditions of drought stress at different hierarchical levels, but to the authors’ knowledge, this is the first study to analyse the genetic variation in drought tolerance including populations, families and clones, that allow a more complete knowledge of the genetic basis of the traits under study. Also, the comparison of results in controlled and field conditions allows the evaluation of the efficiency of early selection method based on PEG-induced osmotic stress for drought tolerance in Maritime pine.

In experimental ecology and genetics, results from tree seedlings and saplings are widely used for extrapolation to possible responses of mature individuals, or to evaluate the most critical phases in the regeneration or recruitment of individuals [6] that are essential for the microevolution of species. Our results provide information which contributes to improve the estimation of levels of genetic variation that are available for micro evolution under drought conditions.

### Evaluations in PEG experiment

The results of the PEG-induced osmotic stress experiment showed the existence of high levels of genetic variation in survival (T50, S100) at the three levels of variation analyzed: population, additive and total genetic variation. Furthermore, these two survival traits are highly correlated at each hierarchical level analyzed, thus S100 can be considered a suitable and easily recorded, estimator of the performance of the different entities, which could be used in future experiments.

In parallel with previous results in this species, *P. pinaster* [19,28,33,38–40], total biomass, root stem biomass allocation and leaf water content, are key determinants for survival at population level under hydric stress in controlled conditions. The higher survival rates were observed for the Spanish-Atlantic provenances, in opposition with the Atlantic-French origins and the Moroccan TAMR, which presented the lowest survival rates. These results are divergent from the ones obtained in field conditions and at more mature ages [21,23,29], with the Spanish Atlantic origins presenting lowest survival rates under stressed environments, in comparison to the Mediterranean ones with clear differences among the Portuguese and French Atlantic provenances (see a more detailed discussion in the following section). We observed contrasting allocation strategies among the Atlantic populations. This pattern has also been described in seedlings of these same populations [31]. 

The Moroccan population (TAMR) is the one with the lowest values of T50 and survival, although it has been reported as a drought tolerance population. In our experiment this population presented a large inversion of root biomass (data not presented) and higher values of LWC and RWC. Even though the size of plants was previously normalized for both aerial and root part, and in the experiment control treatment the survival rate was 100% in all genotypes, this results could be related to a slower rhizogenesis process of this origin in comparison to clones from other sources (the lowest value for root biomass). Effectively, mortality increased rapidly in the first stage of the experiment and maintained in the second part.

The ability to osmoregulate could be related to geographic origin; generally, genotypes from dryer regions exhibiting faster and deeper osmotic regulation when subjected to drought [19]. However, in this work, survival under PEG-induced osmotic stress conditions is not related to any climatic variable of the population (results not shown).

Contrary to the results obtained at population level, relative water content presented a positive additive genetic correlation with survival traits (α<0.05), indicating that, under these controlled experimental conditions, osmotic adjustment may have a major role in drought tolerance than biomass allocation or vigor (measured by aerial biomass). 

RWC can be considered as an integrated measure of the plant water status and is an indicator for the metabolic activity in leaf tissues. RWC has long been considered as a useful screening tool in relation to drought tolerance of crops [41] as it is considered an integrated measure of plant water status and indicator of the metabolic activity in leaf tissues [42]. These results clarify the role of osmotic adjustment in drought tolerance in maritime pine families and clones, suggesting it to be a more important factor than changes in biomass allocation, as has been found in other pines (e.g. Canary pine [15]).

### Relationship between PEG-induced osmotic stress experiment and field performance

The drivers of population performance are highly correlated, independent of experimental design, as indicated by the clear relationship between survival in the xeric field trial and T50 and S100, as well as between these two traits and growth at the mesic site. Furthermore, height growth is highly correlated (p<0.001) between the two sites both at the population and additive level (cross-environmental correlations), indicating a high level of constraint for the evolution of this trait under different environments [35].

The results obtained in this study shows that populations with higher growth present better survival rates, particularly marked in the Spanish Atlantic provenances. This result accords with other studies performed with genetic material of similar pedigree to that used in this study although under very different conditions [17,25,28,29]. In Maritime pine, Corcuera et al. [20] found that populations from more mesic origins displayed plasticity for growth and increased height in more favorable environmental conditions, but a population originated from a drier climate showed low plasticity for growth in both trials. Also, Ritcher et al. [13], showed that *Pinus* from southern provenances are less plastic than continental ones. In the case of Mediterranean *P. sylvestris* and *P. nigra*, it might be an adaptation to regular summer drought in order to prevent futile investment into aboveground structures during seasons of high resource availability [43]. Basically, high resource investment into belowground structures limits a species capacity for aboveground growth optimization in years with moderate drought events [44], which are still likely to occur regularly in temperate forests under climate change.

The results obtained support those of previous, independent studies showing that the Morocco provenance exhibited reduced physiological activity and high growth stability in all the locations tested while the Atlantic populations reacted quickly to interannual and special climatic variability [27]. Young *P. pinaster* trees have also been shown to produce biomass despite low water availability, although root growth seems to stop in order to improve the resources to the photosynthetic system [45]. Conversely, Corcuera et al. [12] found that the xeric populations did not reduce their root surface area in response to drought, while the others populations did.

### Within population variation

The potential for adaptive evolution of quantitative traits depends on the amplitude of their additive genetic variance (measured by heritability) as well as the genetic covariance (correlation) between traits [46]. All the analyzed traits in this study display an intermediate to high heritability, as found in previous studies where high levels of additive genetic variance for biomass traits have been reported [28], as well as for cavitation resistance [33]. Comparing our results for broad and narrow sense heritabilities, non-additive effects appear to be of little importance for survival and growth under either controlled or field conditions. Consequently, the ability exists not only to generate genetic diversity, but also adaptive genetic diversity, and thereby evolve through natural selection. 

Natural selection may have different intensities in different populations, leading to local adaptation, or might shape the level of genetic variance for various traits. A similar interpretation has been proposed for other species [47].

Within populations, survival conditioned by osmotic adjustment was not related to biomass production, allocation or even to survival and growth under mesic or xeric conditions. Hence the implications of our results for the adaptive evolution of the species are mainly based on the high level of standing variation and in the lack of constraints at clone and individual level for the adaptation to different contrasting environments. Diversifying or uniform selection (i.e. [33]) have been invoked to explain different drought related traits in maritime pine. Due to the levels of differentiation found in most cases in comparison to neutral expectations [33,48,49], the lack of tradeoffs (no negative genetic or additive correlations), and the reduced level of non-additive genetic effects in survival and growth traits, the observed pattern is a by-product of selection at population level, and do not respond to the adaptation of populations to drought. As such, local adaptation seems to play a minor role for those traits, contrary to the results obtained in cork oak, which has similar environmental constraints [47]. 

### Application to early evaluation for drought tolerance

The higher values obtained for population correlations in comparison with the values found for additive and total genetic correlations suggests that population is the more determinant factor in the adaptation of genetic materials, and that other mechanisms may be more important at family and clonal level. The wide range in the ability of the provenances examined to survive under contrasting conditions suggests that selection for improving drought tolerance of maritime pine forestry is indeed possible. In fact, the high correlations observed between survival parameters evaluated in the PEG trial, and height and survival in the field trials support the notion that drought testing with PEG is suitable for early selection of populations for drought resistance. Selection when trees are as young as possible, using artificial environments, where tighter control of conditions is possible, is of extreme importance to tree breeding programs. The main constraint on the management of breeding populations of forest trees is the length of time required to complete an entire cycle of breeding, testing and selection, which can range from several years to decades [5]. Moreover, the efficiency of selection for drought tolerance must be increased by identifying site factors or morpho-physiological traits that are closely associated with increased growth under drought. Factors related to biomass allocation and morphology, as well as needle gas exchange measurements will be important for the screening of maritime pine seedlings for drought tolerance, hence further research at a more advanced ontogenetic stage of the plants is needed.

This study contributes to clarify the role of experimental evaluation under controlled conditions in the genetics of drought tolerance by improving understanding of its relationship with field performance. Based on the results we conclude that non additive effects are of scarce importance for drought tolerance in *P. pinaster*, and demonstrate that different strategies are employed at population and within population level; while osmotic adjustment plays an important role in within population variation, biomass allocation and water content seem to greatly determine population level differences in survival. 

## Materials and Methods

### Ethics Statement

*Pinus pinaster* is not a protected or endangered species and therefore, not specific permissions were required for collecting the seed lots. Permissions required for field trials were obtained from INIA and SERIDA.

### Plant material

Ten autochthonous populations were selected along a latitudinal cline: two French Atlantic populations (MIMI and PLEU), four Spanish Atlantic populations (ARMY, CDVO, PTOV, SCRI), two Central Spanish population (ASPE, COCA), one Southern Spanish population (ORIA) and one Moroccan population (TAMR). Seeds from five mother trees were collected within each population, resulting in fifty open pollinated families. Mother trees were separated from each other by a distance of more than 50 m in order to avoid inbreeding [50]. Details on the provenances used in this study are presented in Table 4. 

**Table 4:** Location and climatic information of the studied populations.

**Code**	**Provenance**	**Origin**	**Latitude**	**Longitude**	**Altitude (m)**	**Rainfall (mm)**	**Mean annual temperature (°C)**
**ARMY**	Asturias	Northern Spain	43°18’N	6°29’W	532	1160	11.8
**ASPE**	Ávila	Central Spain	40°12′N	5°3′W	728	1318	14.2
**CDVO**	Asturias	Northern Spain	43°32’N	6°25’W	180	1316	13.2
**COCA**	Segovia	Central Spain	41°15’N	4°30’W	780	454	12.3
**MIMI**	Mimizán	Landes France	44°08’N	1°18’W	37	995	13.8
**ORIA**	Almería	South Spain	37°31′N	2°21’W	1150	357	15.8
**PLEU**	Pleucadec	Landes France	47°46′N	2°20’W	80	855	12.0
**PTOV**	Asturias	Northern Spain	43°33’N	6°38’W	98	1283	13.4
**SCRI**	Pontevedra	Northwest Spain	42°07’N	8°21’W	727	1600	12.3
**TAMR**	Tamrabta	Morocco	33°36′N	5°01′W	1760	763	10.8

Five half-siblings from each of the five families from each of the 10 populations were clonally propagated (using the protocol described in [37]), in order to produce mother plants for subsequent vegetative propagation. Orthotropic cuttings (3-5 cm in length, which is suitable for use as stem mini-cuttings) were collected from the hedged stock mother plants in early spring and set into trays for rooting. For both glasshouse and field experiments, the cuttings were grown for 4 months in 200 mL containers containing peat and perlite (70:30, v/v) and fertilized weekly with the same solution used for the mother plants.

Depending on rooting success and after completing a plant inventory we tested 204 clones (10 provenances and a minimum of 4 families and 4 half sibs per family). 

### PEG-induced osmotic stress experiment in controlled conditions

A nested experimental design with 3 completely randomized blocks was used. The hydric stress was induced by lowering the osmotic potential of the nutrient solution until -1 MPa, using the osmotic agent (polyethylene glycol 8000 - PEG8000) according to [51]. The cuttings were introduced in hydroponic culture by means of a floating system and maintained in an aerated nutrient solution: 4 mequiv L^-1^ NO_3_^-^; 0.38mequiv L^-1^ PO_4_H^2-^; 1.08 mequiv L^-1^ SO_4_^2-^; 1.97 mequivL^-1^ K^+^; 2.41 mequiv L^-1^ Ca^2+^; 1.08 mequiv L^-1^ Mg ^2+^and 25 mg L^-1^ commercial micronutrient mixture (Hortriline, Compo Spain) at pH 6, resulting in an osmotic potential of -0.05MPa. The experiment was performed in polystyrene boxes containing a volume of 120 L of nutrient solution. After reaching the desired potential, the plants remained in the osmotic solution for 7 months. The progression of the osmotic potential of the solution was monitored every 2-3 weeks using a psychrometer (Thermocouple C52 chamber, Wescor, USA). The experiment was performed in the SERIDA glasshouse (Asturias, Spain, 43° 23´N 6° 4´W at 60 m a.s.l). 

### Field trial experiments

A randomized complete block design (CR) with 8 blocks and single-tree plots was considered the baseline experimental design for each trial. Clones were planted in 2009 with square spacing (2.4 m x 0.7 m) resulting in 1,632 trees per site. Selection of experimental design and statistical analysis can yield considerable improvements in terms of heritability, precision of predicted genetic values and genetic gain from selection [5]. We used a single-tree plot, which increases the correlation between true and predicted clonal values, allows more effective sampling of environmental variation and reduces error variance to a greater extent than other tree plot designs [52]. Two replicates of this trial were set up, one in a Mesic site in Grado, Asturias (Northern Spain) and one in a Xeric site in Madrid (Central Spain). The climatic characterization of both sites is described in Table 5.

**Table 5:** Climatic characterization of the field trials (Mesic site- Grado, Xeric site-Madrid).

**Trial Site**	**Period**	**T**	**TM**	**Tm**	**R**	**H**	**DR**	**DF**	**DH**	**I**
**Mesic site-Grado**	**Spring**	11.30	15.63	7.00	95.0	77.0	12.0	8.0	0.3	152.3
	**Smmmer**	17.70	21.83	13.50	53.3	80.3	8.0	11.7	0.0	171.0
	**Year**	12.90	17.00	8.80	973.0	78.0	122.0	100.0	8.0	1711.0
**Xeric site-** **Madrid**	**Spring**	12.37	18.87	5.80	36.3	57.7	5.7	0.3	2.3	232.0
	**Summer**	23.13	31.20	15.03	16.3	42.7	2.7	0.0	0.0	323.7
	**Year**	14.10	20.60	7.60	386.0	59.0	58.00	19.0	54.0	2658.0

**T**: Mean temperature (°C). **TM**: Mean of maximum daily temperature (°C), **Tm**: Mean of minimum daily temperature (°C). **R**: Rainfall (mm). **H**: Average relative humidity (%). **DR**: Number of days with precipitation greater or equal to 1 mm. **DF**: Number of foggy days. **DH**: Number of days with frost. **I**: Number of sunshine hours. (Climate dataset includes a minimum of 23 years).

### Variables measured and analysed

In the PEG-induced osmotic stress experiment under controlled conditions, survival was monitored every working day. At the end of the experiment, plants of the genotypes with at least 3 surviving replicates were harvested. For each harvested plant, the dry biomass (root and shoot components) was measured and the shoot biomass ratio (SBR—shoot biomass/total plant biomass, g g^−1^), root biomass (ROB- root biomass, g) and total biomass (TB, g) were subsequently derived. 

Immediately after each harvest, 1g of needles were weighed (fresh weight - FW), then placed in water to obtain their fresh weight at full hydration (FW100), and finally dried at 70°C for 48 h to obtain their dry weight (DW). Relative water content [RWC= (FW- DW) / (FW100 – DW)] and leaf water content [LWC= (FW-DW)/FW] were then calculated.

In the field trial experiments, 1 year after plantation and for the two field tests (coded M: Mesic site in Grado, X: Xeric site in Madrid), survival (M_SV, X_SV respectively) and total height (M_HT, X_HT) were evaluated. 

### Statistical analysis

#### Survival analysis under PEG-induced osmotic stress experiment

To estimate the proportion of plants surviving at a given time, and hence the survival probability at that time for each clone, the Kaplan-Meier method [53] was used as a product-limit estimator [54]. This principle makes it possible to work with conditional and cumulative probabilities. The conditional survival probability for the generic time interval (the condition being that if the subject was surviving at the beginning of the interval, it must have been surviving in all previous intervals too) is given by:

$$p_{i}=\frac{r_{i}-d_{i}}{r_{i}}$$ [1]

Where *r* is the number of plants alive, hence at risk, at the beginning of the *i* ^th^ interval, and *d* the number of failures during the same interval (time expressed in days). Survival at any time point is calculated as the product of the conditional probabilities of surviving each previous time interval.

$$S(t)=\prod_{i\leq t}\left(1-\frac{d_{i}}{r_{i}}\right)$$ [2]

Comparisons of the different experimental groups were made using the log-rank and Wilcoxon test. Using the Kaplan-Meier method and LIFETEST procedure, comparisons of survival over the entire curve, and not only at the end can be made between different provenances.

Various time intervals were considered to establish the most useful for further analysis and the two most effective were identified as: survival at 100th day after the beginning of the experiment (S100), as this is when the differences between populations were at their highest, see results); and the number of days required by each genotype to reach 50% of deaths as a consequence of the stress applied (T50).

### Quantitative genetic model

Genetic analysis was conducted with the following mixed model:

$$Y_{jpicn}=\mu +B_{j}+P_{p}+F_{i(p)}+C_{c(i)}+\varepsilon_{jpicn}$$ [3]

where: y is the individual phenotypic observation, μ, the overall mean of the variable; B_j_, the effect of the j^th^ block (fixed), P_p_, the effect of the p^th^ provenance (fixed); F_i(p)_, the i^th^ family within the p^th^ provenance and C_c(i)_ is the i^th^ clone within the family. This model was established assuming that the residuals were independent and normally distributed (0, Ve) for the fowling variables: T50, S100, M_HT, X_HT, LWC, RWC, SBR, ROB, TB. For M_SV, X_SV we followed a GLMM model with the same structure and a binomial logit link function. Differences between populations means were evaluated using Duncan test for the continuous variables and a nonparametric comparison for each pair using Wilcoxon method for the binomial variables. A α-value ≤0.05 was taken as statistically significant.

Variance components for family within population (σ^2^_f_ ), clone within family (σ^2^_c_) and residual error (σ^2^_ε_), with the respective associated standard errors, were estimated by restricted maximum likelihood, using the average information REML algorithm implemented in the ASREML programme [55]. We extracted the population effect to estimate the additive and total genetic variation within population, in order that all families and clones were considered as belonging to the same population. 

Additive variance (σ^2^_a_) was estimated assuming that seedlings from the same family were half-sibs [56]. 

 Narrow sense (h^2^) and Broad sense (H^2^) heritabilities were calculated for each trait as

$$h^{2}=\frac{\sigma_{a}^{2}}{\sigma_{P}^{2}}=\frac{4\times \sigma_{f}^{2}}{\sigma_{f}^{2}+\sigma_{c}^{2}+\sigma_{\varepsilon }^{2}}$$ [4]

$$h^{2}=\frac{\sigma_{G}^{2}}{\sigma_{P}^{2}}=\frac{\sigma_{a}^{2}\times \sigma_{c}^{2}}{\sigma_{f}^{2}+\sigma_{c}^{2}+\sigma_{\varepsilon }^{2}}$$ [5]

and standard errors for heritability were estimated by ASREML using a Taylor series approximation [55]. For the two binomial traits (M_SV and X_SV), heritability estimates had to be converted to the liability scale for comparison across trials using the following relationship [57].

$$h_{L}^{2}=h_{0/1}^{2}=\frac{p(1-p)}{z^{2}}$$ [6]

where h^2^_0/1_ is the heritability on the observed binominal scale, h^2^_L_ is the heritability for survival on the underlying (liability) scale, *p* is the incidence of survival in the trial, and *z* is the height of the ordinate at the threshold corresponding to the incidence in that trial.

Pearson’s correlation coefficients were estimated between provenance Best Linear Unbiased Estimator (BLUE) values, between families Best Linear Unbiased Predictor (BLUP). We will refer to these as population correlations and additive genetic correlations respectively. For the total genetic correlations, all Pearson’s correlations were computed over the BLUP family plus BLUP clone.

## Supporting Information

Table S1 **Log-rank test and Wilcoxon test on the survival rates for the different provenances.**
(DOCX)

Table S2 **Variance components obtained from the mixed model.**
(DOCX)
