# Supplementary material for: Genetic Variation of Drought Tolerance in Pinus pinaster at Three Hierarchical Levels: A Comparison of Induced Osmotic Stress and Field Testing
Source: PLoS One. 2013 Nov 1;8(11):e79094. doi: 10.1371/journal.pone.0079094 (PMC3815124; doi:10.1371/journal.pone.0079094)
Supplement: Table S1 — Log-rank test and Wilcoxon test on the survival rates for the different provenances. (DOCX) [file pone.0079094.s001.docx]

**Supporting Information**

**Table S1.** Log-rank test and Wilcoxon test on the survival rates for the different provenances.

| **Test** | **Χ^2^** | **df** | **Significance (α)** |
| --- | --- | --- | --- |
| Log-rank | 126.9 | 9 | < 0.0001 |
| Wilcoxon | 177.4 | 9 | < 0.0001 |
