# Supplementary material for: Genetic Variation of Drought Tolerance in Pinus pinaster at Three Hierarchical Levels: A Comparison of Induced Osmotic Stress and Field Testing
Source: PLoS One. 2013 Nov 1;8(11):e79094. doi: 10.1371/journal.pone.0079094 (PMC3815124; doi:10.1371/journal.pone.0079094)
Supplement: Table S2 — Variance components obtained from the mixed model. (DOCX) [file pone.0079094.s002.docx]

**Supporting Information**

**Table S2.** Variance components obtained from the mixed model.

|  | V_f_ | V_c_ | V_e_ |
| --- | --- | --- | --- |
| T50 | 87.48 ** | 93.01 * | 933.13 |
| S100 | 70.82 *** | 98.60 *** | 627.25 |
| M_HT | 1.85*** | 4.25 *** | 25.66 |
| X_HT | 197.82 *** | 347,60*** | 2485.63 |
| M_SV | -ns | 0.64*** | 3.29^a^ |
| X_SV | 0.155 * | 0.24 * | 3.29^a^ |

Abbreviations as in Table 1;V_f_: family variance; Vc: clonal variance; V_e_: error variance. Statistical significance: *:α < 0.05, **: α < 0.01; ****α* < 0.001; ns: non significant. ^a:^ values corrected for a binomial logit link function.
